# Is the exposure to bisphosphonates or osteoporosis the predictor of spinal radiographic progression in ankylosing spondylitis?

**DOI:** 10.1186/s13075-018-1730-9

**Published:** 2018-10-18

**Authors:** Orsolini Giovanni, Adami Giovanni, Rossini Maurizio, Fassio Angelo, Giollo Alessandro, Caimmi Cristian, Idolazzi Luca, Gatti Davide, Viapiana Ombretta

**Affiliations:** University of Verona, Rheumatology Unit, piazzale L. Scuro 10, 37134 Verona, Italy

We read with great interest the paper by Deminger et al. [1] reporting determinants of radiographic progression in ankylosing spondylitis (AS). Intriguingly, the authors found bisphosphonate exposure during follow up to be a predictor of spinal radiographic progression in women. Correctly, the authors declared that their results are based on few observations and should be interpreted with caution. Indeed, it is known that the prevalence of osteoporosis, the main indication for bisphosphonate use, is high in AS patients [2] and associated with radiographic progression of the disease [3].

Unfortunately, the study presented lacks data on osteoporosis prevalence, bone mineral density, vertebral fractures, or bone turnover markers in the patients enrolled. Considering the pathophysiology of the disease, there’s rationale for a positive rather than negative effect of bisphosphonates. First, they lower bone resorption, which is known to be elevated at least in the early stages of AS. Second, suppression of bone resorption by bisphosphonates would lead, through the coupling of osteoclast and osteoblast activity, to later inhibition also of bone formation and to an increase in serum sclerostin [4], an inhibitor of the anabolic bone Wnt pathway and which has been described to be low in AS and negatively correlated to the development of syndesmophytes [5].

Although interesting, the conclusion of the authors might send an alarming message on bisphosphonate use in AS that could worsen an already low treatment rate of osteoporosis and vertebral fracture in those patients, complicating their disability. In our opinion the hypothesis that the association observed was due to more severe bone loss in those patients who show radiographic progression and that leads to the use of bisphosphonates should be considered and further investigated.

## Abbreviation

AS: Ankylosing spondylitis
