# Correction to: Is the exposure to bisphosphonates or osteoporosis the predictor of spinal radiographic progression in ankylosing spondylitis?

**DOI:** 10.1186/s13075-018-1753-2

**Published:** 2018-11-05

**Authors:** Giovanni Orsolini, Giovanni Adami, Maurizio Rossini, Angelo Fassio, Alessandro Giollo, Cristian Caimmi, Luca Idolazzi, Davide Gatti, Ombretta Viapiana

**Affiliations:** University of Verona, Rheumatology Unit, piazzale L. Scuro 10, 37134 Verona, Italy

## Correction

Following publication of the original article [1], the authors reported a typesetting error. The author’s given names and family names have been inverted. The correct author panel is as follows:

Given name: Giovanni, Family name: Orsolini

Given name: Giovanni, Family name: Adami

Given name: Maurizio, Family name: Rossini

Given name: Angelo, Family name: Fassio

Given name: Alessandro, Family name: Giollo

Given name: Cristian, Family name: Caimmi

Given name: Luca, Family name: Idolazzi

Given name: Davide, Family name: Gatti

Given name: Ombretta, Family name: Viapiana

The publishers apologise for this error. The original article [1] has been corrected.
